# “You can’t see what you’ve never had to live”—Cultivating imagination and solution spaces in global health and development

**DOI:** 10.1371/journal.pgph.0005242

**Published:** 2025-09-30

**Authors:** Safieh Shah, Shruti Bora, Esme Supriya Gupta Longley, Erika Valtierra, Madhukar Pai

**Affiliations:** 1 Institute for Globally Distributed Open Research and Education, Karachi, Pakistan; 2 Generation Mental Health, India; 3 School of Population and Global Health, McGill University, Montreal, Canada; 4 Center for Global Health, Partners in Health, Lima, Peru; PLOS: Public Library of Science, UNITED STATES OF AMERICA

Elon Musk, the world's wealthiest man, recently declared on X (Twitter) that *“The path to solving hunger, disease and poverty is AI and robotics”* [1]*.* He did not mention taxing the rich as a potential solution. Musk’s power and privilege limit or shrink his imagination, for he cannot see what he has never had to live.

Musk’s statement reflects a deeper systemic truth–that the most visible “solutions” to hunger, poverty, and disease are often imagined by those who have been structurally insulated from scarcity, dispossession or violence. Because they never have to face the consequences of their own band-aid solutions, their imaginations are not shaped by real stakes in the matter. Furthermore, they can worsen our shared realities by monopolising which futures are considered “realistic” or worthy. It is no surprise that Musk led the efforts to defund United States (U.S.) government aid and science organizations, and worsened hunger and disease [2].

## How and why solution spaces shrink

Ideally, our space to imagine ought to be unconstrained. Similarly, when we seek solutions, we need to begin by considering all possible ways of doing things. Often, this is structurally challenging in global health.

Global health and development fields are almost invariably led by cisgender, heterosexual men, Global North experts, and their local elite counterparts in the Global South [3]. This concentration of power often reflects entrenched hierarchies rooted in patriarchy, caste, race, class, ethnicity, language, ableism, and historical power structures. This inherent lack of diverse representation, whether in Global North boardrooms or among Global South elites, renders these fields highly prone to elite capture [4].

Elites, however competent or compassionate, are conditioned by patriarchal and racial capitalist systems that prevent them from seeing what they cannot see and what they have not had to live through. This results in them prioritizing issues based on their own privileges, granting them access to all sorts of spaces and decision-making roles simply because of their whiteness, passport power, or wealth. This problem is reflected in global leadership today, which is predominantly male and increasingly authoritarian. Their worldview often promotes wars and conflicts, instead of societal causes. Toxic male leadership in politics has reversed progress in gender and reproductive rights.

Consequently, Black, brown, Indigenous, young, and marginalized communities without these generational privileges struggle to even be heard in the very spaces that are intended to “help” them. They are not seen as producers of knowledge but seen as “beneficiaries” of elite wisdom, which results in epistemic injustice, and warped spaces of imagination.

As an example, in 2001, a USAID administrator claimed that HIV antiretroviral therapy (ART) for Africans would be challenging because *“[Africans] do not know what watches and clocks are”* [5]. With one ignorant statement based on his limited worldview, an American expert dramatically shrunk the solution space for the AIDS crisis in Africa. In contrast, African activists demanded faster ART access, not just access to condoms or abstinence advice.

## Solution spaces shrink or grow depending on who we center

The Covid-19 pandemic offers another example, where Covax was designed by Global North partners to get vaccines to low-income countries. While Covax did deliver vaccines, it failed to anticipate vaccine hoarding by rich nations and Big Pharma’s greed in preferentially supplying wealthy buyers [6]. What if Covax had been designed by experts in the Global South? They would have wanted less reliance on trickle-down charity, and instead demanded an intellectual property waiver, vaccine technology transfer, and Global South-based manufacturing [7]. While Global North solutions typically involve charity, solutions from the Global South more often focus on self-determination and self-reliance [8].

Following the U.S. withdrawal from the WHO in 2025, and the collapse of major aid initiatives, many Global North experts wrote about how to resurrect aid. However, since these authors often failed to include voices of the so-called “beneficiaries” of aid, their solutions often recapitulated charity-based models, or called for more U.S. leadership [9]. In contrast, non-elite Global South experts are reimaging aid by raising concerns about power dynamics, harmful consequences of the Western aid model, and the need for greater self-reliance by Global South governments [10].

It’s deeply ironic that as the climate crisis intensifies, privileged elites gather at conferences like Davos and COP to devise solutions whilst simultaneously worsening the problem. Many arrive in private jets, contributing to the very issue they claim to solve. Unsurprisingly, these elites seem conditioned not to see how extractivism and infinite growth models which sustain their wealth actually worsen the climate crisis and associated health outcomes. They pitch technocratic solutions or put the onus on individual action but skirt the central issue of their own carbon footprint and tax avoidance [11]. They pre-emptively shape the imaginative landscape, making justice-centred solutions seem “unthinkable” [11]. In contrast, Global South leaders have proposed solutions like debt relief for climate-stricken nations and a “loss and damage” fund as climate reparations [11].

**Fig 1:**
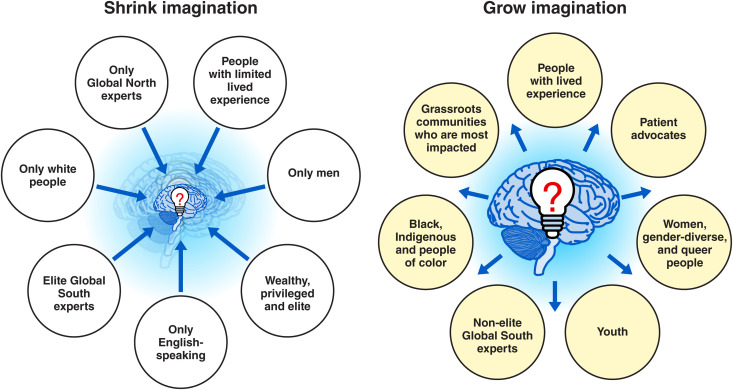
Solution spaces expand or shrink depending on who we engage and center. They grow (right panel) when we center on the expertise of people with lived experience and who are the most impacted by structural violence, such as; patient advocates, women and gender-diverse people, youth, non-elite Global South experts, Black, Indigenous and people of color, queer people, people with disabilities, non-anglophone, and grassroots communities who are most impacted. Conversely, they shrink (left panel) when we center only men, elite, privileged, wealthy, only English-speaking, only Global North experts, or elite Global South experts.

These case studies show us how solution spaces (Fig 1) can expand or shrink depending on who we engage and center and who gets to lead. With diversity, equity and inclusion (DEI) efforts increasingly under attack, it’s worth emphasizing the dangerous consequences of not having diversity and inclusion—it dramatically shrinks our collective imagination and thus, solution spaces. Thus, we risk making huge mistakes.

Our ability to open our own imagination is conditional on the effort we put in. When we are not intentional, we may invite only men or Global North or anglophone experts to meetings,and miss people with the most lived experience. With the growing anti-immigrant sentiment in many regions, hosting events in visa-hostile, Global North settings may shrink solution spaces, by excluding people with vital lived experiences [12].

Similarly, while young people lead many social justice and public health movements such as gun control, abortion rights, anti-genocide protests, climate justice, their engagement is often tokenised. In reality, some of the most important work on climate justice comes from the youth [11]. Their moral clarity and their imagination are less constrained than that of world leaders beholden to fossil fuel companies and endless economic growth models [11]. But tokenistic youth inclusion perpetuates when we refuse to acknowledge co-option and elite capture among youth.

The same principle is evidenced in efforts to reduce Black infant and maternal deaths in the U.S. The most meaningful work comes from Black-led midwifery and reproductive justice movements that center the voices of Black birthing people as a form of expertise [13]. Again, effective solutions originate from the communities they directly impact, because they are the real experts. As cancer survivor and social justice advocate Kwanele Asante wrote, *“lived experience must be considered a form of expertise.”* [14].

## Shifting power, building people power

These are not new ideas; they show how kindness and empathy are the highest forms of intelligence. From historical peoples’ movements (e.g. civil rights, gay rights, anti-apartheid, women's suffrage, AIDS activism), we know change rarely comes from powerful people or political leaders. It invariably comes from ‘people power,’ which requires organizing, and restoring people to the center of political processes to achieve real change.

This idea of ‘people power’ is echoed by contemporary scholars like Seye Abimbola, who argues for recentering and localizing global health on people with less power (‘the periphery’) [15], and patient advocates who emphasize the critical need for the healthcare system to listen to patients [14].

Allowing people’s voices into decision-making spaces would mean ceding power, disrupting profit, and re-centering priorities around justice rather than extraction. Expanding imagination would therefore require redistributing epistemic power; not just ‘inviting’ representation of lived experience but shifting the authority to define problems and design solutions to those most affected by these issues. Otherwise, the periphery will remain the periphery.

So, the next time we reimagine global health, write a paper, or plan a conference or a commission, we must ask ourselves: Is this our work to do, are we truly growing the collective imagination here? Are we the right people to lead this or should we step back and hold space as allies or co-liberators for others instead? Have we assembled the right expertise by centering those who are most vulnerable and knowledgeable? When we intentionally ask these questions and act on the answers, we will unlock truly hidden solutions and find real leaders. As Indigenous people, patient advocates, people living with disabilities, as well as AIDS activists often remind us: “*Nothing about us, without us*.”

## References

[1] The News International. Elon Musk makes bold statement with new AI plan; 2025 [cited 31 Aug 2025]. Available from: https://www.thenews.com.pk/latest/1332238-elon-musk-makes-bold-statement-with-new-ai-plan

[2] Goldberg M. Elon Musk’s legacy is disease, starvation and death New York Times; 2025. [cited 5 Aug 2025]Available from: https://www.nytimes.com/2025/05/30/opinion/elon-musk-doge-usaid.html

[3] Global Health 50/50. The Global Health 50/50 report 2022: Boards for all? London, UK; 2022 [cited 9 Jan 2023]. Available from: https://globalhealth5050.org/2022-Report/

[4] Krugman DW. Global health and the elite capture of decolonization: on reformism and the possibilities of alternate paths. PLOS Glob Public Health. 2023;3(6):e0002103. doi: 10.1371/journal.pgph.0002103 37384634 PMC10309605

[5] Attaran A. Adherence to HAART: Africans take medicines more faithfully than North Americans. PLoS Med. 2007;4(2):e83. doi: 10.1371/journal.pmed.0040083 17326715 PMC1808103

[6] Pushkaran A, Chattu VK, Narayanan P. A critical analysis of COVAX alliance and corresponding global health governance and policy issues: a scoping review. BMJ Glob Health. 2023;8(10):e012168. doi: 10.1136/bmjgh-2023-012168 37793808 PMC10551961

[7] Kyobutungi C, Gitahi G, Wangari M-C, Siema P, Gitau E, Sipalla F, et al. From vaccine to visa apartheid, how anti-Blackness persists in global health. PLOS Glob Public Health. 2023;3(2):e0001663. doi: 10.1371/journal.pgph.0001663 36963085 PMC10021597

[8] Pai M, Bandara S, Kyobutungi C. Shifting power in global health will require leadership by the Global South and allyship by the Global North. Lancet. 2024:S0140-6736(24)02323-7. doi: 10.1016/S0140-6736(24)02323-7 39491869

[9] Sebelius K. Without the United States, global health will fall apart; 2025 [cited 5 Aug 2025]. Available from: https://www.nytimes.com/2025/01/24/opinion/who-trump-us-global-health.html

[10] Kyobutungi C, Okereke E, Abimbola S. After USAID: what now for aid and Africa? BMJ. 2025;388:r479. doi: 10.1136/bmj.r479 40068845

[11] Deivanayagam TA, Osborne RE. Breaking free from tunnel vision for climate change and health. PLOS Glob Public Health. 2023;3(3):e0001684. doi: 10.1371/journal.pgph.0001684 36963098 PMC10021701

[12] Bandara S, Deng N, Pai M. The Global North is increasingly unsafe for global health meetings. Lancet. 2025;405(10491):1728–30. doi: 10.1016/S0140-6736(25)00757-3 40324446

[13] Opara IN, Elmi YM. Reimagining Black maternal health narratives: embracing a vitality framework for joy, liberation, and healing. PLOS Glob Public Health. 2025;5(7):e0004703. doi: 10.1371/journal.pgph.0004703 40680103 PMC12273923

[14] Asante K, Chinegwundoh F, Hammonds RM. Decolonisation of global health must include civil society. BMJ. 2025;390:r1264. doi: 10.1136/bmj.r1264 40628451 PMC12231275

[15] Abimbola S. The Foreign Gaze. IRD Editions; 2025 [cited accessed 5 Aug 2025]. Available from: https://www.editions.ird.fr/produit/728/9782709930437/the-foreign-gaze

